# The *Eucommia ulmoides - Achyranthes bidentata* pair and their active monomers exert synergistic therapeutic potential for osteoarthritis through the PI3K-AKT pathway

**DOI:** 10.3389/fphar.2025.1571884

**Published:** 2025-08-11

**Authors:** Chun Chen, Lei Lv, Yueying Huang, Fangyuan Xie, Mingzhu Gao, Sailiang Zeng, Zijun Wang, Xue Jiang, Yangyang Zhan, Leilei Bao

**Affiliations:** ^1^ Department of Pharmacy, Third Affiliated Hospital of Naval Medical University, Shanghai, China; ^2^ Jiangxi University of Traditional Chinese Medicine, Nanchang, China

**Keywords:** drug pair, osteoarthritis, synergies, Pi3k-akt, apoptosis

## Abstract

**Background:**

Osteoarthritis is characterized by articular cartilage degradation, involving inflammation-mediated chondrocyte apoptosis and extracellular matrix destruction. Eucommia ulmoides and Achyranthes bidentata constitute a classic herbal pair for OA treatment, yet their combinatorial effects and molecular mechanisms remain unelucidated.

**Methods:**

The EU-AB extract was prepared via aqueous decoction. An LPS-induced ATDC5 chondrocyte inflammatory model and an MIA-induced rat OA model were established. Therapeutic efficacy was evaluated using Lequesne scores, ELISA, Western blotting, and immunohistochemistry. Bioactive components were identified by HPLC-TOF/MS, while RNA-seq and molecular interaction analyses validated underlying mechanisms.

**Results:**

The EU-AB extract significantly suppressed the expression of matrix metalloproteinases (MMP-3/13) and inflammatory cytokines (NO, TNF-α, IL-6, IL-1β) in both ATDC5 cells and rat serum (*P* < 0.05). Concurrently, it reduced Lequesne scores and joint swelling in MIA-induced OA rats (*P* < 0.05) while ameliorating histopathological cartilage damage. Among 35 compounds identified by HPLC-TOF/MS, pinoresinol diglucoside (PIN) from EU and chikusetsusaponin Ⅳa (CHI) from AB demonstrated synergistic effects, downregulating pro-apoptotic proteins (Caspase-3/9, Bax) through activation of the PI3K-Akt pathway and promotion of Akt phosphorylation.

**Conclusion:**

The herbal pair aqueous extract suppresses osteoarthritis via the bioactive component group CHI-PIN, demonstrating synergistic anti-inflammatory effects in MIA rats, likely mediated by PI3K-Akt-regulated apoptosis.

## Graphical Abstract

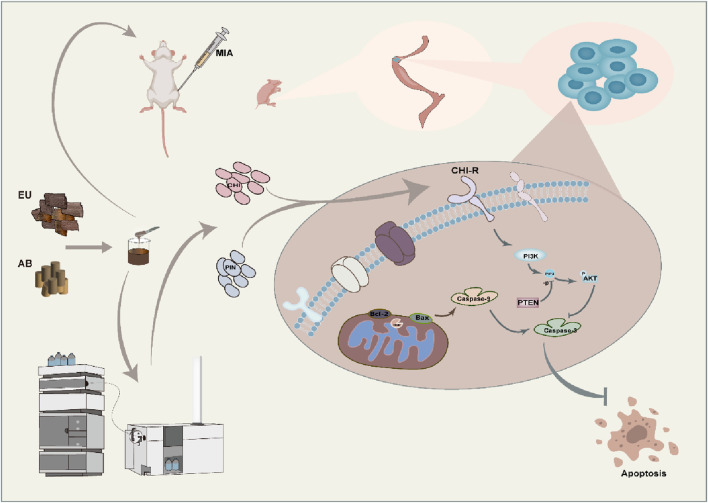

## Introduction

OA affects over 500 million people globally, characterized by progressive cartilage degradation, subchondral bone remodeling, and synovial inflammation (Abramoff and Caldera, 2020). This leads to debilitating pain, joint dysfunction, and significant socioeconomic burden (Colletti and Cicero, 2021). Conventional pharmacotherapies (NSAIDs, corticosteroids) offer symptomatic relief but carry substantial side effects with limited disease-modifying efficacy (Liang et al., 2022).

Traditional Chinese Medicine (TCM) has gained attention for its holistic approach, particularly herb pairs like EU and AB (Zhou et al., 2023). The *EU-AB* originates from “The Couplet Medicines of the Traditional Chinese Medicine Encyclopedia”. Combining the above can multiply the power of tonifying the liver and kidneys and strengthening the muscles and bones. EU demonstrates chondroprotective effects through PI3K-Akt-mediated suppression of IL-1β, IL-6, and RANKL/OPG imbalance (Huang et al., 2021; Wang et al., 2016; Chong et al., 2022). AB exerts its effects by inhibiting the action of MMP-3 and MMP-13 (Zhao et al., 2021). EU and AB can likely exert anti-OA effects by inhibiting cell apoptosis and exerting anti-inflammatory actions (Zhang et al., 2021; Zhang et al., 2020). Moreover, their active components have been proven to effectively promote bone formation (Yang et al., 2020), angiogenesis (He et al., 2023), and stimulate tissue development and regeneration from various perspectives.

Clinical formulations containing EU-AB (e.g., Duhuo Jisheng decoction) show efficacy, yet mechanistic validation of their synergistic interaction remains absent despite network pharmacology predictions of multi-target effects (Jian et al., 2020). However, whether the pair can treat OA as expected and the key molecular mechanisms have yet to be experimentally verified.

In previous studies, we confirmed the EU-AB have synergistic anti-inflammatory effect on RAW264.7 cells (Gao et al., 2022), analyzed the crucial components in pairs and explored the reasons for the changes in the content after their combination (Chen et al., 2024). This article further clarifies the anti-OA effects of the pair, uncovers the key components that contribute to the synergistic effect, and anchors the related pathways for regulation, providing insights into the potential molecular mechanisms underlying the synergistic therapeutic effects of the pair on OA.

## Methods

### Material

EU (Batch No.: 191231) and AB (Batch No.: 210326) were procured from the Shanghai Hongqiao Traditional Chinese Medicine Slices Company, and voucher specimens were stored in the Pharmacy Department of the Third Affiliated Hospital of Naval Medical University. EU is the dried bark of Eucommia ulmoides Oliv., and AB, belonging to the family Amaranthaceae, is the dried root of Achyranthes bidentata Bli. Celecoxib capsules (CL) were bought from Sichuan Guowei Pharmaceutical Co., Ltd., and Tenghuang Jiangu capsules (TH) were acquired from Gansu Xifeng Pharmaceutical Co., Ltd. ELISA kits for MMP-3, MMP-13, NO, iNOS, IL-1β, IL-6, and TNF-α were received from Jiangsu Meimian Industrial Co., Ltd. Sodium iodate was sourced from Sigma and the Total RNA Kit for total RNA extraction was procured from Yeasen Biotechnology Co., Ltd. Gefitinib (GEF) and dexamethasone (DEX) were obtained from Shanghai Macklin Biochemical Technology Co., Ltd. The standard substances CHI and PIN were supplied by Shanghai Yuanye Biotechnology Co., Ltd., and the proteins Akt, p-Akt, Bax, Caspase-3, and Caspase-9 were obtained from CST.

### Drug preparation

Take 100 g EU and 100 g AB. Add water at 1:10 ratio to soak overnight in a beaker. The next day, heat the solution to 100°C and maintain for 1 h. Repeat this process 3 times. Filter, prepare extract paste, store at −20°C. Dilute with water to required concentration before use. For single herbs: take 200 g EU or 200 g AB, prepare using same method as herb-pair water extract. CL and TH were dissolved in purified water to achieve their respective target concentrations. All treatments were administered via intragastric administration for 4 weeks, with drug-specific doses detailed in Table 1. The concentration for monomer groups was experimentally determined to be 10^−6^ M.

**TABLE 1:** Drug doses in the 4-week intragastric treatment protocol.

Drug	Dosage
TH	0.1714 g/kg*d
CL	0.0171 g/kg*d
EU	1.2 g/kg*d
AB	1.2 g/kg*d
P-Low	1.2 g/kg*d
P-Middle	2.4 g/kg*d
P-High	4.8 g/kg*d

### Animal modeling and grouping

Sprague-Dawley (SD) rats (200 ± 20 g) of specific pathogenfree grade were supplied by Shanghai Regen Biological Co., Ltd. (experimental animal production license number: SCXK 2019-0002). All SD rats were kept in the animal room of the School of Pharmacy of Naval Medical University, under a fixed room temperature of 20°C and a 12-h light/dark cycle. The experiment initially randomly assigned 72 rats into the following 9 groups: control, model, CL, TH, EU, AB, low-dose pair (P-L), medium-dose pair (P-M), and high-dose pair (P-H). Each group contained 8 rats with sex-balanced distribution. However, due to attrition unrelated to the research protocol, the final sample size per group was reduced to 6 rats. Except for the control group, the remaining eight groups underwent model construction: after anesthesia with 2% pentobarbital and waiting for about 5 min until the rats had no limb reflexes. The right hind limbs were bent to 90°. Using an insulin syringe, 50 µL of MIA was slowly injected into the joint cavities of rats. The needle was slowly withdrawn, and fluid seepage was checked (Chun et al., 2021).

### Assessment indicators

Measurements of the following indicators were performed on days 0, 7, 14, and 21. The joint diameter of the affected limb was measured by wrapping a cotton thread around the swollen area of the rat joint (Abdel-Rahman et al., 2020). Electric von Frey was used to determine the mechanical pain threshold (Liao et al., 2020), and behavioral scoring was used to assess the rats’ responses to pain, gait changes, range of motion of the joints, and joint swelling in four aspects (Shi et al., 2020). The scoring table can be found in Table 2.

**TABLE 2:** Standard of Lequesne MG scores.

Items	Behavioral manifestation of animals	Scores
Local pain reaction through palpation	No abnormal pain response	0
	Mild contraction of affected hind limb	1
	Contraction of affected hind limb with systemic reaction, such as shaking, turning head to lick	2
	Severe contraction of affected hind limb with shaking, struggling or escaping	3
Gait changes	Normal gait without limping	0
	Mild limping when running	1
	The affected hind limb could participate in walking but limped obviously	2
	The affected hind limb couldn’t participate in walking, touch or pedal the ground	3
Range of motion of affected joint (Straight position = 0°)	Above 90°	0
	45°∼90°	1
	15°∼45°	2
	Below 15°	3
Degree of joint swelling	No swelling, bone markers clearly visible	0
	Mild swelling, bone markers shallow	1
	Obvious swelling, disappearance of bone markers	2

### Histological evaluation

After the fourth week of drug administration, all rats were decapitated and killed, the right knee joints were fixed in 4% paraformaldehyde, and sections were stained with H&E and Safranin O-Fast Green staining to assess histological differences (Chun et al., 2021). The most severe areas of each section were selected and scored using the OASRI and the Modified Mankin’s score (Cai et al., 2020).

### Immunohistochemistry

The sections were then processed for immunohistochemistry. After incubation with 10% goat serum at room temperature for 30 min, 50–100 µL of MMP-3/13 antibody was added and incubated overnight at 4°C. Following TBST washing, the secondary antibody was added. After color development, counterstaining, drying, and mounting, the state of the joint sections was observed under a microscope (Xie et al., 2023).

### Enzyme-linked immunosorbent assay (ELISA)

During the second week of the experiment, blood was collected from rats via the orbital sinus. Blood samples were left at room temperature for 2 h and then centrifuged at 3,500 rpm for 10 min to obtain the supernatant. The serum levels of NO, IL-1β, MMP-3, MMP-13, TNF-α, and IL-6 were measured following the instructions provided with the ELISA kits.

### HPLC-MS method

The mobile phase comprises 0.1% formic acid in water (A) and acetonitrile (B). The system was equilibrated for 3 min at a flow rate of 0.5 mL/min with a column temperature of 25°C and an injection volume of 2 μL. The gradient elution program was as follows: 0–10 min, 15%–30% B; 10–20 min, 30%–70% B; 20–30 min, 70% B. The TOF mass spectrometer was equipped with an AJS-ESI source, operating in positive and negative ion modes, with a sheath gas temperature of 350 °C, a drying gas flow rate of 10 L/min, a nebulizer pressure of 40 psi, a fragmentor voltage of 160 V, and a capillary voltage of 3500 V. Aligent 6,470 Series QQQ Triple quad mass spectrometry (Aligent, United States) was applied in mass spectrometry, Mass spectrometric detection was using dynamic multiple response monitoring (dMRM) mode equipped with AJS-ESI source in the negative ion mode. The parameters were set as follows: nebulizer gas temperature 325°C, drying gas flow rate 10 L/min, nebulizer pressure 40 psi, sheath gas temperature 350°C, sheath gas flow rate 11 L/min, capillary voltage 3500 V, nozzle voltage 1500 V, and nitrogen as the collision gas.

### Cell isolation and differentiation

Newborn fetuses were euthanized for subsequent experiments. The fetuses were disinfected with 75% ethanol, and the hind limb skin was cut open using surgical scissors. Muscle tissue was removed as much as possible. The hind limbs were collected, placed in a culture dish, and washed several times with pre-cooled PBS. They were digested for 60 min with 2 mg/mL trypsin-EDTA solution, washed several times with PBS, and digested for 90 min with 3 mg/mL collagenase D solution. The digestion was continued overnight with a 0.5 mg/mL collagenase D solution. The next day, once complete digestion was achieved, the fragments were repeatedly blown with a pipette gun, filtered through a 40 µm sterile filter, and centrifuged at 300 × g for 5 min. After washing with PBS and centrifugation, cells were resuspended in DMEM and transferred to a new culture dish. The cell culture was observed daily, and the culture fluid was replaced on the third day (Lu et al., 2023). ATDC5 murine embryonic carcinoma cells were bought from iCell Bioscience Inc.

### Cell membrane chromatography construction

Approximately 10^7^ ATDC5 cells were harvested and cultured on ice. Then PBS was added to wash thrice. The cells were scraped off, sonicated at 400 W every 10 s, and centrifuged at 1,000 rpm for 10 min to remove the supernatant. Centrifugation was performed again at 12,000 rpm for 20 min to obtain the cell membrane pellet resuspended in 5 mL physiological saline. Silica gel was added to react and form the stationary phase of the cell membrane and rotated overnight at 4 °C; then, the column was packed the next day. The flow conditions for the packed cell membrane column were as follows: from 0 to 5 min, a flow rate from 0.2 to 1.0 mL/min; from 5 to 5.5 min, 1.0 mL/min. When the pressure stabilized, the cell membrane chromatography column was packed. The mobile phase was 10 mM ammonium acetate, with a flow rate of 0.5 mL/min, a column temperature of 37°C, and an injection volume of 5 µL (Chai et al., 2022).

### Genome sequencing

Differential gene expression was determined after drug treatment in a primary chondrocyte model. RNA sequencing and data analysis were performed using Genewiz (China). Total RNA was extracted from primary mouse chondrocytes using a Total RNA Kit. RNA quality was assessed with the Agilent 2,100 Bioanalyzer, and further procedures were implemented only once the RNA met the required standards. The collected RNA samples were subjected to library construction and sequencing. The general steps were as follows: mRNA was captured using magnetic beads with poly T probes and fragmented at high temperatures using a magnesium ion solution. Random primers were used to synthesize double-stranded cDNA, and the ends were repaired using the dA tail reagent. Polymerase chain reaction amplification was performed using P5 and P7 primers, followed by validation. The samples were then loaded onto an Illumina HiSeq/Illumina Novaseq/MGI2000 instrument for sequencing. Data analysis was conducted using R language scripts.

### Immunoblotting assay

Proteins obtained after treatment with radioimmunoprecipitation assay lysis buffer were separated by sodium dodecyl sulfate-polyacrylamide gel electrophoresis and transferred onto a polyvinylidene fluoride membrane. After blocking at room temperature for 60 min, the membrane was incubated with the primary antibody overnight. After washing with TBST, the secondary antibody was added and incubated for 1 h. The membrane was developed and photographed using an imaging system.

### Evaluation of synergistic effect

The effect of combining two single drugs/compounds was evaluated using King’s formula: $Q=\frac{EAB}{EA+EB-EA*EB}$ (Variables: EA, EB: Observed inhibition rate (%) of single agents; EAB: Measured inhibition rate of the drug combination). When Q < 0.85, it was considered that the combination of two drugs produces an antagonistic effect. When 0.85 < Q < 1.15, the drug pair produces an additive effect. When Q > 1.15, the two drugs were considered to have a synergistic effect (Wang et al., 2018).

### Statistical analysis

The experimental data were processed using GraphPad Prism software (version 8.0). Except for OARSI and Mankin scores, which were analyzed using non-parametric tests, all other data were evaluated by one-way ANOVA followed by Tukey’s HSD post hoc test for multiple comparisons, and Welch’s ANOVA was applied to part of the data. Details can be found in the Supplementary Material. A difference was considered significant when *P < 0.05* and highly significant when *P < 0.01*. The results compared with the blank control group are indicated by #, while those compared with the model group are indicated by *.

## Results

### Inflammatory response inhibition in ATDC5 cells

**FIGURE 1:**
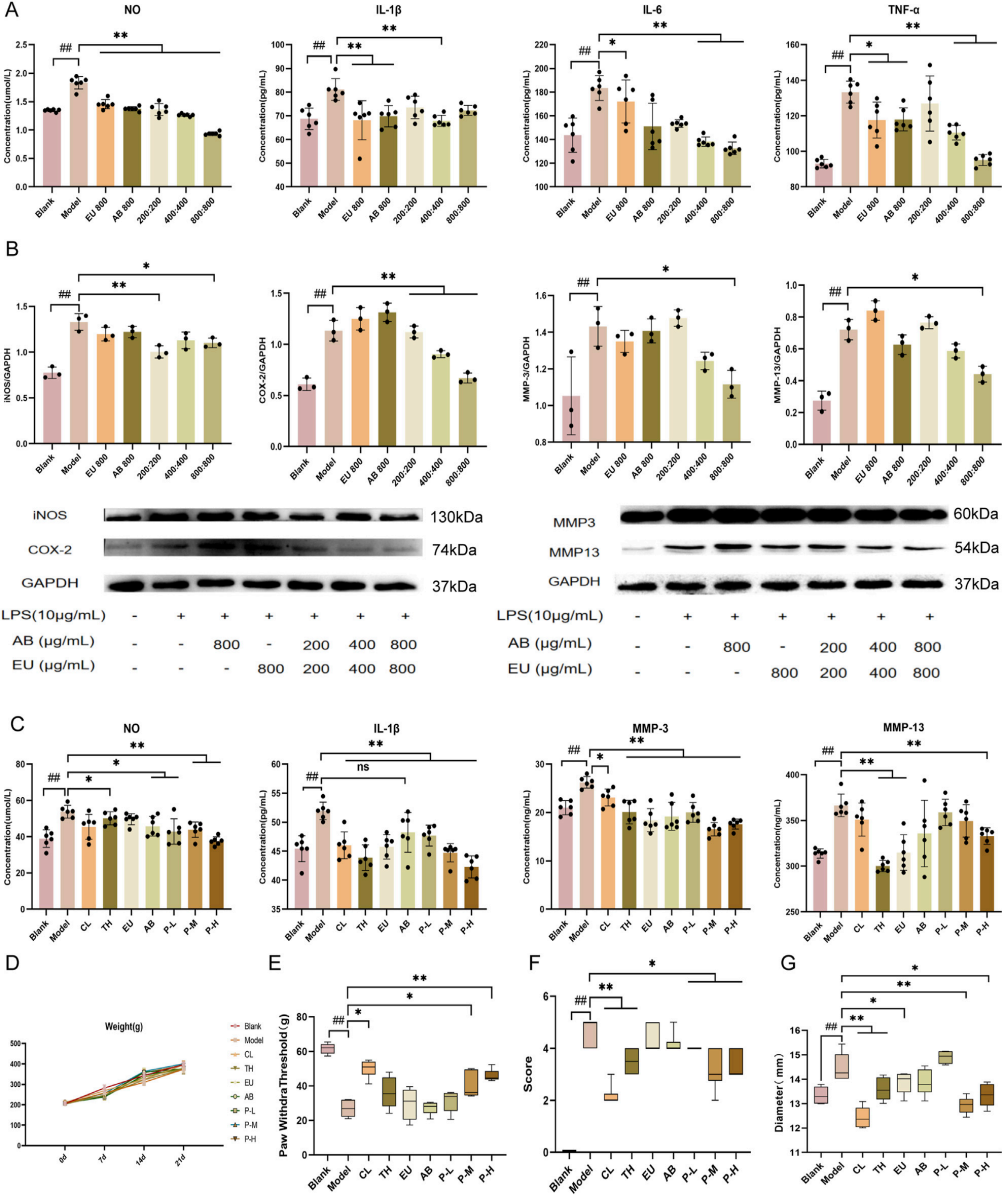
Inhibition of inflammatory response by the aqueous extract of the pair and improvement of behavior in OA rats. **(A)** Expression of NO, IL-1β, MMP-3, and MMP-13 in the supernatant of ATDC5 cells. **(B)** Expression of proteins iNOS, COX-2, MMP-3, and MMP-13 in ATDC5 cells. **(C)** Expression of iNOS, COX-2, MMP-3, and MMP-13 in the serum of rats. **(D)** Changes in body weight of rats within 21 days of administration. Changes in mechanical pain threshold **(E)**, behavioral score **(F)**, and joint diameter **(G)** after intervention with the herbal pair in model rats. Data are expressed as mean ± SD.

To determine the effect of the aqueous extract of the pair on inflammation inhibition, ATDC5 cells were examined. The ATDC5 cells used in the experiment were differentiated into chondrocytes after 21 days of stimulation with ITS. LPS can induce an inflammatory response in ATDC5 cells and increase the expression of the MMP family. After intervention with 10 μg/mL LPS for 24 h, the levels of the inflammatory factors NO, IL-1β, IL-6, and TNF-α in the cell supernatant increased (Figure 1A, *P < 0.01*), confirming the successful establishment of the model. Intervention with *EU* and *AB* alone inhibited the trend of increased inflammatory factors (*P < 0.01*), and the same inhibitory effect on inflammatory factors was found in the medium and high-dose groups of the pair (*P < 0.01*). Subsequently, protein expression in ATDC5 inflammatory model cells was measured after intervention with single herbs and the pair (Figure 1B). The expression of iNOS, COX-2, MMP-3, and MMP-13 proteins was significantly increased in the model group (*P < 0.01*). The single herbs of *EU* and *AB* did not exhibit a significant inhibitory trend, while the high-dose group, after combined use, downregulated the expression of iNOS, COX-2, MMP-3, and MMP-13 proteins (*P < 0.05*).

### Inhibition of inflammatory responses in OA rats

The blank group was not intervened and was intragastrically administered an equal dose of physiological saline during the experimental period. In the *in vivo* experiment, the CL and TH groups were used as positive controls. All rats simulated the pathological characteristics of OA after intra-articular injection of MIA, which was manifested by increased levels of NO, IL-1β, MMP-3, and MMP-13 in the serum, reduced mechanical pain threshold, knee swelling, and a significant decrease in the animal behavior score according to the Lequesne scoring (Figures 1C–G) after modeling. It was found that the pair group could also reverse this trend in the rat model, and through the observation of joint diameter, mechanical pain threshold, and behavior in rats, the swelling was reduced, the pain was relieved, and the behavior was improved after modeling. Moreover, the body weight of all experimental groups of rats after 3 weeks of intragastric administration was non-significantly different from that of the normal group, and drug intervention did not cause severe adverse reactions in the rats.

### Protection of joints in OA rats by the pair

**FIGURE 2:**
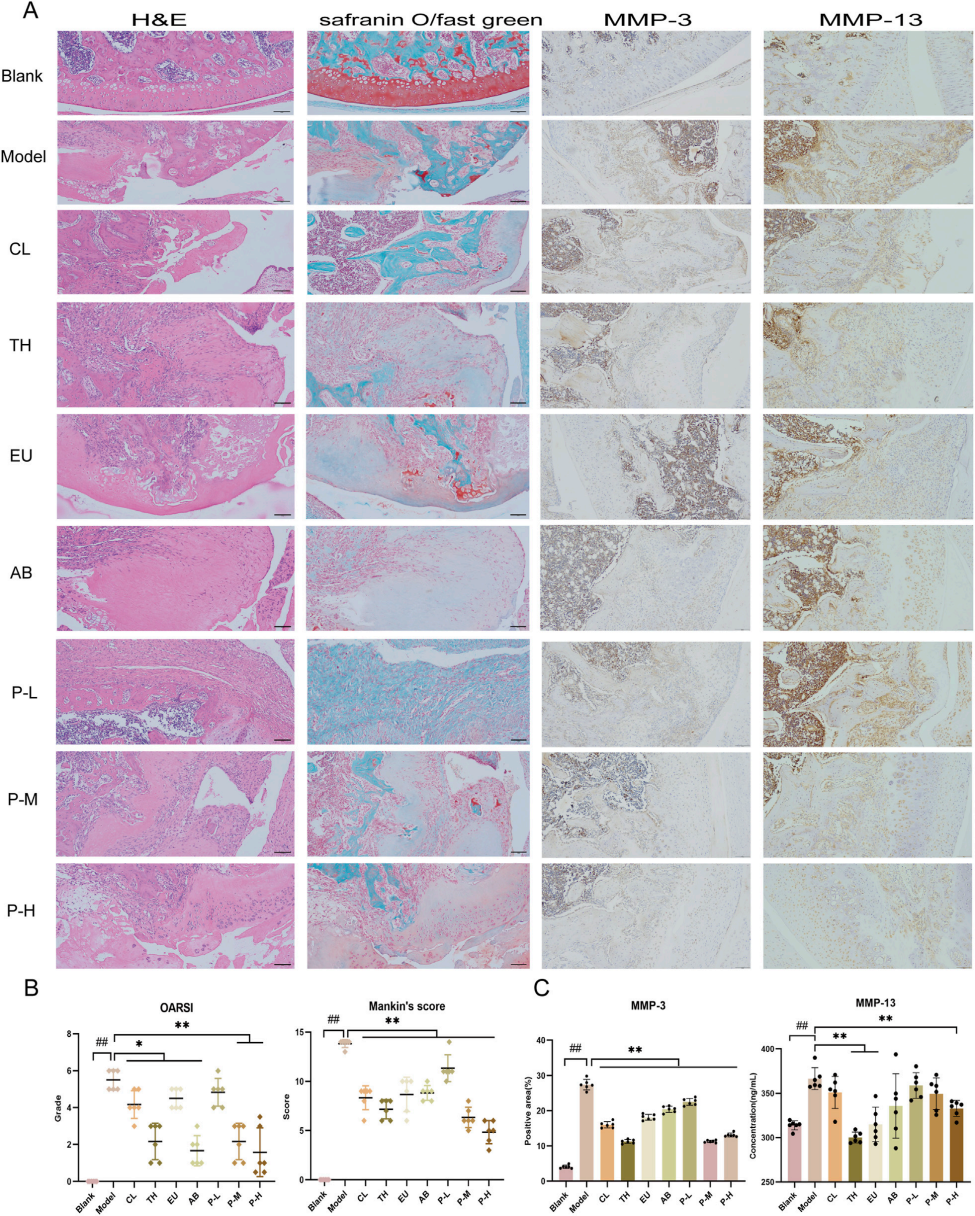
The pair inhibits cartilage matrix degradation and delays the pathological process in joints. **(A)** From left to right are the HE, Safranin O, and immunohistochemical staining for MMP-3 and MMP-13 in the knee joints of rats after the intervention. **(B)** OARSI grading and Mankin’s scoring of articular cartilage. **(C)** Expression of immunohistochemical MMP-3 and MMP-13 in each group. Data are expressed as mean ± SD.

HE and Safranin O staining of the pair group depicted a certain degree of repair effect on the articular cartilage (Figure 2A). The cartilage structure was relatively intact, with cracks only on the surface, which did not affect the entire structure, and the staining degree of the cartilage matrix was relatively light. According to the staining results, the joints of each group were graded and scored, and the OARSI grade and Mankin’s score of the pair group decreased. In the immunohistochemical results of MMP-3 and MMP-13 (Figure 2A), the pair group significantly inhibited the production of proteases and reduced cartilage degradation.

### Synergistic effect of the aqueous extract against OA

The synergistic effects of the aqueous extract on behavior, mechanical pain, joint diameter, cytokine levels, and immunohistochemistry were calculated using King’s formula by comparing the effects obtained from the medium-dose group of the pair with those of the single herb (Figure 2B). The results are presented in Table 3. On the 7th day, the herbal pair illustrated a synergistic effect in relieving pain. On the 14th day, the effect on improving behavior was significant, and on the 21st day, it synergistically alleviated the affected limb swelling. Additionally, immunohistochemical results indicated that the herbal pair synergistically inhibited the expression of MMP-3 on the articular cartilage surface (Figure 2C).

**TABLE 3:** Synergistic effect evaluation of the aqueous extract against OA.

Indicator	EU (efficacy rate/inhibition rate,%)	AB (efficacy rate/inhibition rate,%)	P-Middle (efficacy rate/inhibition rate,%)	Q
Behavioral score				
7 d	3.85	3.85	7.69	1.02
14 d	7.14	10.71	32.14	1.88
21 d	8.33	20.33	37.5	1.37
Mechanical pain threshold				
7 d	9.35	1.2	33.01	3.16
14 d	18.13	16.49	35.6	1.13
21 d	23.38	29.78	27.42	0.59
Joint diameter				
7 d	4.12	5.64	5.71	0.60
14 d	7.52	6.94	13.22	0.95
21 d	4.27	4.25	10.65	1.28
Cytokine				
NO	7.68	14.93	18.48	0.86
MMP-3	29.84	26.82	36.95	0.76
MMP-13	14.08	8.4	4.64	0.22
IL-1β	11.95	7.15	13.91	0.76
Immunohistochemistry				
MMP-3	33.87	25.57	58.74	1.16
MMP-13	46.80	39.36	54.16	0.80

### CHI has an affinity for ATDC5 cell membranes

**FIGURE 3:**
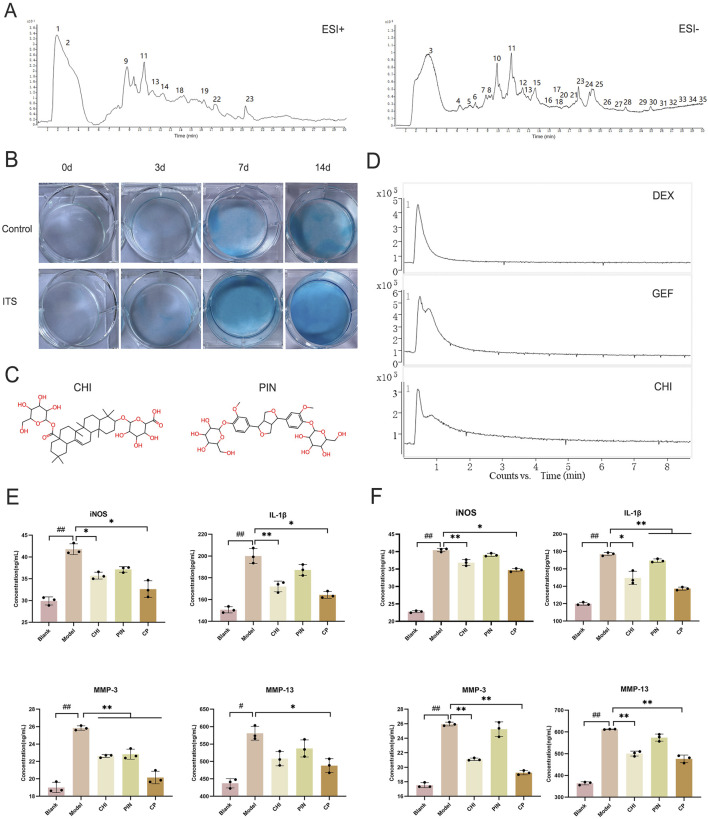
The effective component group inhibited the inflammatory response in ATDC5 cells and primary chondrocytes. **(A)** Total ion chromatogram of the herbal pair aqueous extract in positive and negative ionization modes. **(B)** Degree of differentiation of primary chondrocytes at 0, 3, 7, and 14 days with and without ITS. **(C)** Chemical structures of CHI and PIN. **(D)** Retention of DEX, GEF, and CHI on the chondrocyte membrane chromatography column. Expression levels of iNOS, IL-1β, MMP-3, and MMP-13 in the supernatant of ATDC5 cells **(E)** and primary chondrocytes **(F)** after intervention with single compound and compound groups. Data are expressed as mean ± SD.

EU and AB were mixed in a 1:1 ratio, ground to a suitable particle size, and then extracted by soaking, boiling, and filtering to obtain the filtrate. After freeze-drying, the aqueous extract was obtained in syrup form. Before injection into the liquid phase system, the extract was dissolved in water to a 0.02 g/mL concentration. A total of 35 compounds were detected in the crude aqueous extract of the pair in positive and negative ion modes (Figure 3A). The details of each compound are listed in Table 4. These included 8 lignans, 7 phenylpropanoids, 4 steroidal compounds, 7 saponins, 2 alkaloids, 4 cycloartane ether compounds, and 3 others. Among the nine monomers that entered the bloodstream, the CHI from AB depicted retention behavior on the chondrocyte membrane chromatography column (Figure 3D). From EU, the compound PIN was selected to pair with CHI, and the structures of these two monomers are displayed in Figure 3C.

**TABLE 4:** TOF/MS results for the identification of the chemical components in the aqueous extracts of the drug pair.

No.	RT (min)	Compound	Molecule formula	MW(m/z)	Quasimolecular ion peaks	Measured quality (m/z)	Error (ppm)
1	2.139	Amarasterone A	C30H50O7	522.3556	M + Na	545.3443	0.17
2	2.667	Betaine	C5H11NO2	117.0784	M + H	118.0857	4.92
3	3.799	Vanillic acid	C8H8O4	168.0428	M + COOH	213.041	−3.2
4	6.053	3,4-Dihydroxyhydrocinnamic acid	C9H10O4	182.0572	M-H	181.0511	3.99
5	7.515	Chlorogenic acid	C16H18O9	354.0934	M-H	353.0858	4.84
	7.602			354.0945	M + H	355.1016	1.76
6	7.864	Geniposidic acid	C16H22O10	374.1215	M-H	373.1355	−0.56
7	8.875	(E)-Coniferin	C16H22O8	342.1304	M + COOH	387.1281	3.19
8	8.875	Geniposide	C17H24O10	388.1372	M + COOH	433.1355	−0.54
9	8.997	Ethyl caffeate	C11H12O4	208.073	M + H	209.0802	2.45
10	9.975	Caffeic acid	C9H8O4	180.0416	M-H	179.0338	3.81
11	10.372	Pinoresinol 4-O-beta-D-glucopyranoside	C26H32O11	520.1936	M + H	521.2005	1.66
	11.061				M-H	519.1858	1.35
12	12.239	Polypodine B	C27H44O8	496.3015	M + COOH	541.2998	4.82
13	12.986	Pinoresinol diglucoside	C32H42O16	682.2478	M + COOH	727.2452	−0.74
	11.108				M + Na	705.2354	2.02
14	12.248	palmatine	C21H22NO4	352.1533	M + Na	375.1422	4.5
15	13.678	Coniferyl alcohol	C10H12O3	180.0782	M + COOH	225.0765	2.54
16	14.678	Genipin	C11H14O5	226.0837	M-H	225.0765	1.97
17	15.701	2-Propenoicacid,3-(2,3-dihydroxyphenyl)-,ethylester, (2E)-(9CI)	C11H12O4	208.0722	M-H	207.0648	4.32
18	15.924	Syringaresinol-di-O-glucoside	C34H46O18	742.2674	M + COOH	787.2666	−0.14
	14.128				M + Na	765.2576	1.38
19	16.104	Episyringaresinol	C22H26O8	418.1623	M + H	419.1696	1.06
20	17.308	Cycloolivil	C20H24O7	376.1507	M-H	375.1426	3.98
21	17.542	Citrusin B	C27H36O13	568.2135	M-H	567.2077	3.72
22	17.605	22,25-Epoxy-2,3,14,20-tetrahydroxycholest-7-en-6-one	C27H42O6	462.2969	M + H	463.3039	2.7
23	17.859	β-ecdysterone	C27H44O7	480.3087	M + COOH	525.307	−0.06
	20.109					481.3153	1.98
24	19.076	Di-hydrodehydrodiconiferyl alcohol	C20H24O6	360.1569	M + H	405.1549	1.08
25	19.415	Eucommiol	C9H16O4	188.105	M + COOH	187.0977	−0.88
26	20.383	Ginsenoside Ro	C48H76O19	956.4974	M-H	955.4918	0.7
27	21.606	Achyranthoside D	C53H82O25	1118.515	M-H	1117.5077	−0.12
28	22.493	Chikusetsusaponin IV	C47H74O18	926.4883	M-H	925.4809	−0.88
29	24.228	Chikusetsusaponin IVa	C42H66O14	794.4471	M-H	793.4411	−2.38
30	25.056	1-Octen-2-ol (9CI)	C8H16O	128.1197	M-H	187.1335	3.27
31	26.007	Achyranthoside C	C47H72O20	956.463	M-H	955.4563	−1.39
32	27.757	β-D-Glucopyranosiduronic acid, (3β)-17-carboxy-28-norolean-13 (18)-en-3-yl 3-O-β-D-xylopyranosyl- (9CI)	C41H64O13	764.4358	M-H	763.4288	−1.43
33	28.554	Achyranthoside Ⅱ	C41H62O15	794.4094	M-H	793.4018	−0.7
34	28.613	Calenduloside E	C36H56O9	632.392	M-H	631.3851	0.74
35	29.715	Palmitic acid	C16H32O2	256.2403	M-H	315.2546	−0.21

### CHI-PIN inhibited inflammatory responses through the PI3K-Akt pathway

**FIGURE 4:**
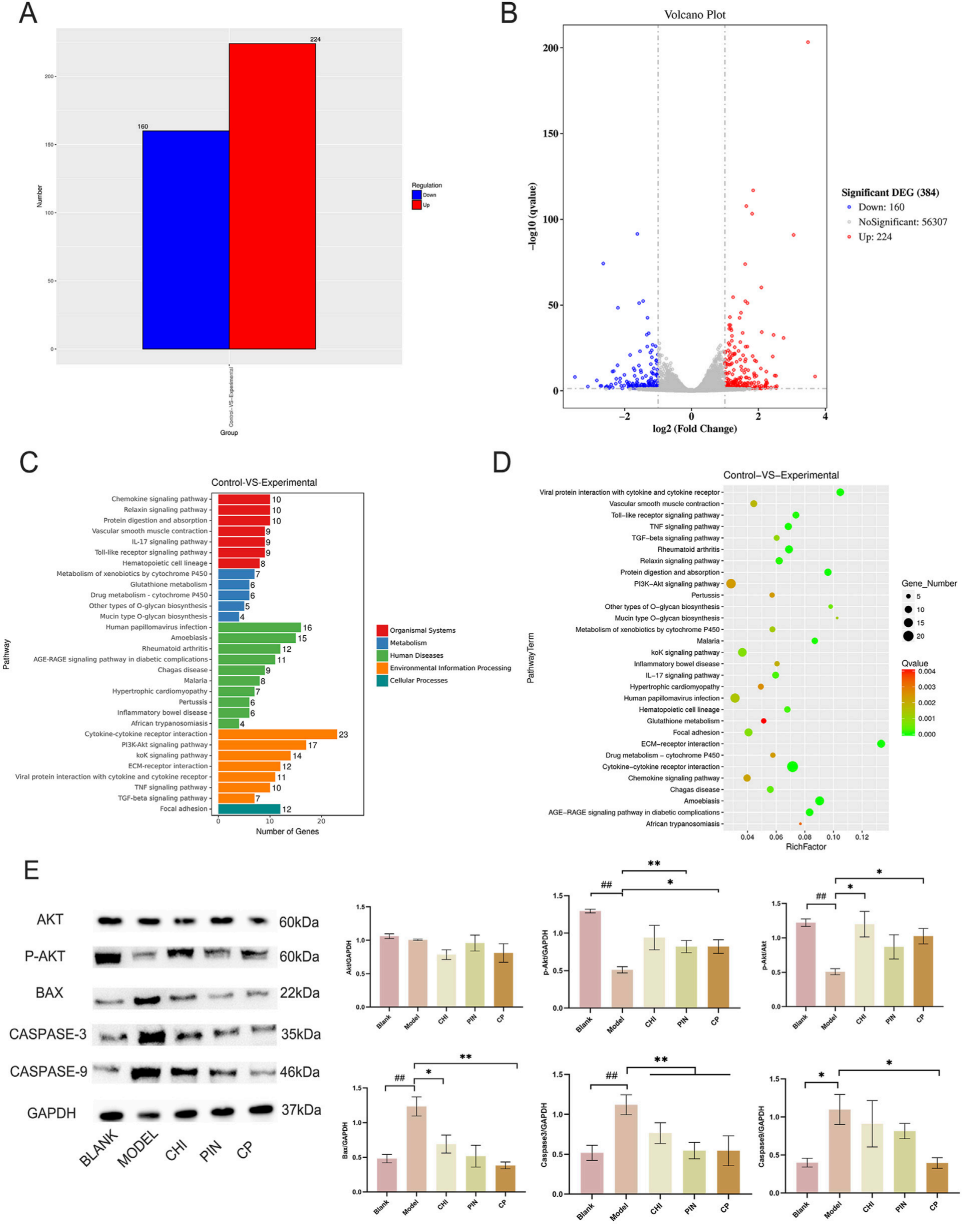
The pair’s key components inhibit apoptosis through the PI3K-Akt pathway. Histogram of differential gene expression between the experimental group after administration and the model group **(A)**, volcano plot **(B)**, KEGG pathway annotation diagram **(C)**, and KEGG enrichment analysis bubble chart **(D)**. Immunoblotting bands and analysis results of Akt, p-Akt, BAX, Caspase-3, and Caspase-9 proteins after intervention with monomers and the monomer group **(E)**. Data are expressed as mean ± SD.

Primary chondrocytes were successfully isolated from suckling mice, and their degree of differentiation was identified using Alcian Blue. Chondrocytes in the third passage contained the most proteoglycans, exhibiting the most obvious degree of differentiation (Figure 3B), consistent with reported results. The monomers suppressed the expression of iNOS, IL-1β, MMP-3, and MMP-13 in ATDC5 cells and primary chondrocytes (Figures 3E,F). This trend was more pronounced after pairing, and by comparing the model group with the treatment group, 384 differential genes were identified, with 160 downregulated genes and 224 upregulated genes in the treatment group compared to the model group (Figures 4A–D). These genes were mainly concentrated in the JAK-STAT, TGF-β, and PI3K-Akt pathways. We selected Akt and p-Akt, key signaling molecules in the classical signaling pathway of OA, the PI3K-Akt pathway, for verification (Figure 4E). Akt expression did not differ significantly among the groups. However, p-Akt depicted a downward trend in the model group, while it was highly expressed in the treatment group. Since the PI3K-Akt pathway is closely related to chondrocyte apoptosis, it was hypothesized that monomers can exert an inhibitory effect on apoptosis through this pathway. To verify this hypothesis, the protein levels of Bax, Caspase-3, and Caspase-9 in the apoptosis pathway were measured, and the treatment group could counteract the increase in these protein levels caused by the model intervention.

## Discussion

The King’s formula method serves as a classical pharmacological framework for evaluating drug synergy. Its validity stems from a pragmatic simplification of the Bliss Independence principle, rendering it particularly suitable for rapid preliminary screening of combinatorial drug potential (Tallarida, 2011). Although the conventional Q-value criterion provides an empirical threshold, it cannot establish probabilistic distribution models. This study preliminarily identifies synergistic tendencies in aqueous herb-pair extracts through this method, aiming to guide further mechanistic investigations. Notably, while the herb-pair aqueous extract combination demonstrated suboptimal synergy in suppressing IL-1β and MMP-13 levels, it exhibited therapeutic potential across distinct OA phases: During the acute phase (7d), the combination may alleviate mechanical allodynia via rapid modulation of ion channels or neuroplasticity. In the chronic phase (21d), joint structural improvements (reduced diameter) likely originate from chondroprotection. This dual-phase efficacy fully satisfies the OARSI guideline efficacy criterion of *'≥50% pain relief + joint structural stabilization'* (Bannuru et al., 2019) – the primary therapeutic target. Critically, drug interactions may evolve temporally; the observed IL-1β/MMP-13 antagonism could reflect compensatory inflammatory activation, warranting long-term safety evaluation.

MIA-induced osteoarthritis models reach pain threshold within 3–7 days (Xu et al., 2020). The herb pair demonstrated optimal synergistic analgesia on day 7, with more pronounced synergistic effects on limb swelling and behavioral improvements during mid-late stages. This indicates that effective pain control in early OA creates conditions for deeper intervention by the herb pair in later phases, while fundamental regulatory effects during mid-late stages consolidate initial efficacy and prevent recurrence (Yunus et al., 2020; Ahmad et al., 2020; Chen et al., 2023). The aqueous extract modulated inflammatory cytokines comparably to positive control groups, with superior inhibition of NO and MMP-3. Studies suggest balanced modulation of NO concentrations can bidirectionally regulate MMP secretion (Prado et al., 2021). When MMPs are inhibited, extracellular matrix is protected from degradation, preventing cellular damage and reducing inflammatory mediator release–establishing a self-reinforcing therapeutic cycle. Our findings reveal the herb pair’s mechanism not only targets upstream inflammatory signaling but also effectively addresses downstream effector molecules (NO) and tissue-destructive matrix metalloproteinases. Therefore, we focus on elucidating how this dual-phase intervention occurs, with primary emphasis on identifying the key active constituents responsible for these effects.

We employed CMC to screen monomers binding to ATDC5 chondrocyte membranes. LPS-induced ATDC5 cells represent a classical osteoarthritis model–when cultured with ITS, they differentiate into mature chondrocytes, offering high reproducibility, cost-effectiveness, and ease of culture. Among nine blood-exposed monomers, CHI bound to ATDC5 membranes. Although no EU components directly bound, certain EU constituents may enhance CHI-membrane affinity synergistically. CHI, a representative triterpenoid saponin from Achyranthes bidentata, effectively suppresses inflammation and bone destruction in rheumatoid mice (Guo et al., 2021) and inhibits IL-6, TNF-α, and IL-10 in RAW264.7 cells (Xu et al., 2021). Elevated CHI levels *in vivo* and *in vitro* post-pairing further suggest EU components facilitate this process. Through *in vitro* validation, the optimal monomer combination was CHI- PIN. PIN, a characteristic EU compound, demonstrates chondroprotective effects in rabbit models (Lou et al., 2022). Individually, both CHI and PIN exhibit anti-OA potential. To validate synergistic anti-inflammatory effects, primary chondrocytes (authenticated by Alcian blue staining) were selected as physiologically relevant models. Monomer proportions mirrored their natural ratios in aqueous extracts to ensure translational relevance. Synergistic efficacy was replicated exclusively with the CHI-PIN combination–other pairings showed inferior effects–indicating their pivotal role in herb-pair synergy. Transcriptomic analysis revealed 39 significantly enriched pathways between OA and CHI-PIN treated groups. Focusing on OA-relevant pathways (Wang et al., 2022), subsequent protein verification confirmed pronounced PI3K-Akt pathway activation following CHI-PIN intervention.

In OA, the degree of degeneration of articular cartilage is closely related to the homeostatic environment of the cartilage and the state of chondrocytes (Sun et al., 2020; Liu Z. et al., 2022). When p67phox is activated, the PI3K-Akt pathway is activated, leading to a significant increase in AKT phosphorylation. On the one hand, this leads to an increase in the expression of Col2a1 and ACAN, promoting the synthesis of the extracellular matrix of chondrocytes to ensure the toughness and compressive strength of cartilage (Liu Z. M. et al., 2022). Contrarily, chondrocytes effectively alleviate the blocking effect of OA in the G0/G1 phase because PTEN is silenced, promoting cell proliferation (Huang et al., 2017; Zhan et al., 2023). Moreover, in IL-1β-induced apoptosis of chondrocytes, activation of the PI3K-Akt pathway can block this process (Zhu et al., 2023; Saeed et al., 2023), as the Bax gene is located downstream of this pathway. When the pathway is activated, it can inhibit the expression of the Bax gene. In our model group, the protein expression of the pro-apoptotic genes Bax, Caspase-3, and Caspase-9 was significantly increased. However, when the intervention was applied to the monomer group, secretion of these proteins was inhibited. CHI-PIN, a regulator of the PI3K-Akt pathway, can be used to treat OA by activating the PI3K-Akt pathway to promote Akt phosphorylation, thereby inducing chondrocyte proliferation and reducing apoptosis to a certain extent to prevent cartilage degeneration and maintain the extracellular matrix in the cartilage to sustain the homeostatic environment of the cartilage. While our mechanistic investigation focused on the PI3K-Akt pathway and confirmed its central role, osteoarthritis pathogenesis involves complex signaling networks. Although there are web-based pharmacological studies pointing to a relationship between the Cortex EU-AB pair and the PI3K-Akt pathway, we have not directly verified whether the whole extract acts through this pathway. In addition, this includes classical inflammatory/catabolic pathways such as NF-κB and MAPK, which exhibit extensive crosstalk with PI3K-Akt. We did not systematically evaluate CHI-PIN’s effects on NF-κB p65 nuclear translocation or MAPK phosphorylation status. Although PI3K-Akt activation sufficiently explains observed protective effects–including anti-apoptotic and pro-anabolic actions–we cannot exclude concurrent modulation of other pathways by CHI-PIN or their potential contribution to the synergistic efficacy.

We declare that during the animal experimentation process, random exclusion was implemented to standardize experimental group sizes following unexpected attrition. We acknowledge that eliminating healthy animals without predetermined criteria risks distorting data distribution. However, retrospective validation confirms the reliability of post-exclusion datasets for core endpoints. We recognize this methodological limitation and commit to implementing rigorous prospective protocols for handling missing data in future studies.

## Conclusion

The aqueous extract of the herbal pair effectively inhibits osteoarthritis progression and demonstrates a synergistic trend in the MIA rat model. Furthermore, our findings strongly suggest that CHI-PIN represents a key bioactive component group within the extract, likely exerting its anti-inflammatory effects by mediating apoptosis through the PI3K-Akt pathway.

## Data Availability

The datasets presented in this study can be found in online repositories. The names of the repository/repositories and accession number(s) can be found in the article/Supplementary Material.

## Abbreviations

OA, Osteoarthritis; EU-AB, Eucommia ulmoides and Achyranthes bidentata; LPS, Lipopolysaccharide; H&E, Hematoxylin and eosin; MMPs, expression of matrix metalloproteinases; NO, nitric oxide; IL, interleukin; TNF-α, tumor necrosis factor-α; iNOS, Inducible Nitric Oxide Synthase; COX, Cyclooxygenase; CHI, chikusetsusaponin Ⅳa; PIN, pinoresinol diglucoside; PI3K-Akt, Phosphatidylinositol-3-kinase-Protein Kinase B signaling; Bax, Bcl-2 Associated X; TCM, traditional Chinese medicine; RANKL, Receptor Activator of Nuclear Factor-κB Ligand; OPG, Osteoprotegerin; PEG2, Polyethylene Glycol 2; CL, Celecoxib capsules; TH, Tenghuang Jiangu capsules; GEF, Gefitinib; DEX, dexamethasone; P-L, low-dose pair; P-M, medium-dose pair; P-H, high-dose pair.
