# Supplementary material for: The Eucommia ulmoides - Achyranthes bidentata pair and their active monomers exert synergistic therapeutic potential for osteoarthritis through the PI3K-AKT pathway
Source: Front Pharmacol. 2025 Aug 11;16:1571884. doi: 10.3389/fphar.2025.1571884 (PMC12375627; doi:10.3389/fphar.2025.1571884)
Supplement: Supplementary file 1 [file Table1.docx]

| The ARRIVE Essential 10 | | | | | |
| --- | --- | --- | --- | --- | --- |
| These items are the basic minimum to include in a manuscript. Without this  information, readers and reviewers cannot assess the reliability of the findings. | | | | | |
| Study design | 1 | | For each experiment, provide brief details of study design including： | | |
|  |  | | a. The groups being compared, including control groups. If no control group has been used, the rationale should be stated. | | 🗹 |
|  |  | | b. The experimental unit (e.g. a single animal, litter, or cage of animals) | | 🗹 |
| Sample size | 2 | | a. Specify the exact number of experimental units allocated to each group, and the total number in each experiment. Also indicate the total number of animals used. | | 🗹 |
|  |  | | b. Explain how the sample size was decided. Provide details of any a priori sample size  calculation, if done. | | 🗹 |
| Inclusion and exclusion criteria | 3 | | a. Describe any criteria used for including and excluding animals (or experimental units) during the experiment, and data points during the analysis. Specify if these criteria were established a priori. If no criteria were set, state this explicitly. | | 🗹 |
|  |  | | b. For each experimental group, report any animals, experimental units or data points not included in the analysis and explain why. If there were no exclusions, state so. | | 🗹 |
|  |  | | c. For each analysis, report the exact value of n in each experimental group. | | 🗹 |
| Randomisation | 4 | | a. State whether randomisation was used to allocate experimental units to control and  treatment groups. If done, provide the method used to generate the randomisation sequence. | | 🗹 |
|  |  | | b. Describe the strategy used to minimise potential confounders such as the order of treatments and measurements, or animal/cage location. If confounders were not contolled, state this explicitly. | | 🗹 |
| Blinding | 5 | | Describe who was aware of the group allocation at the different stages of the experiment (during the allocation, the conduct of the experiment, the outcome assessment, and the data analysis). | | 🗹 |
| Outcome  measures | 6 | | a. Clearly define all outcome measures assessed (e.g. cell death, molecular markers, or behavioural changes). | | 🗹 |
|  |  | | b. For hypothesis-testing studies, specify the primary outcome measure, i.e. the outcome measure that was used to determine the sample size. | | 🗹 |
| Statistical  methods | 7 | | a. Provide details of the statistical methods used for each analysis, including software used. | | 🗹 |
|  |  | | b. Describe any methods used to assess whether the data met the assumptions of the statistical approach, and what was done if the assumptions were not met. | | 🗹 |
| Experimental  animals | 8 | | a. Provide species-appropriate details of the animals used, including species, strain and substrain, sex, age or developmental stage, and, if relevant, weight. | | 🗹 |
|  |  | | b. Provide further relevant information on the provenance of animals, health/immune status, genetic modification status, genotype, and any previous procedures. | | 🗹 |
| Experimental  procedures | 9 | | For each experimental group, including controls, describe the procedures in enough detail to allow others to replicate them, including: | | |
|  |  | | a. What was done, how it was done and what was used. | | 🗹 |
|  |  | | b. When and how often. | | 🗹 |
|  |  | | c. Where (including detail of any acclimatisation periods). | | 🗹 |
|  |  | | d. Why (provide rationale for procedures). | |  |
| Results | 10 | | For each experiment conducted, including independent replications, report: | | |
|  |  | | a. Summary/descriptive statistics for each experimental group, with a measure of variability where applicable (e.g. mean and SD, or median and range). | | 🗹 |
|  |  | | b. If applicable, the effect size with a confidence interval. | | 🗹 |
| The Recommended Set | | | | | |
| These items complement the Essential 10 and add important context to the study.  Reporting the items in both sets represents best practice. | | | | | |
| Abstract | | 11 | | Provide an accurate summary of the research objectives, animal species, strain and sex, key methods, principal findings, and study conclusions. | 🗹 |
| Background | | 12 | | a. Include sufficient scientific background to understand the rationale and context for the study, and explain the experimental approach. | 🗹 |
|  | |  | | b. Explain how the animal species and model used address the scientific objectives and, where appropriate, the relevance to human biology. | 🗹 |
| Objectives | | 13 | | Clearly describe the research question, research objectives and, where appropriate, specific hypotheses being tested. | 🗹 |
| Ethical statement | | 14 | | Provide the name of the ethical review committee or equivalent that has approved the use of animals in this study, and any relevant licence or protocol numbers (if applicable). If  ethical approval was not sought or granted, provide a justification | 🗹 |
| Housing and husbandry | | 15 | | Provide details of housing and husbandry conditions, including any environmental enrichment. | 🗹 |
| Animal care and  monitoring | | 16 | | a. Describe any interventions or steps taken in the experimental protocols to reduce pain, suffering and distress. | 🗹 |
|  | |  | | b. Report any expected or unexpected adverse events. | 🗹 |
|  | |  | | c. Describe the humane endpoints established for the study, the signs that were monitored and the frequency of monitoring. If the study did not have humane endpoints,  state this. | 🗹 |
| Interpretation/  scientific  implications | | 17 | | a. Interpret the results, taking into account the study objectives and hypotheses, current theory and other relevant studies in the literature. | 🗹 |
|  | |  | | b. Comment on the study limitations including potential sources of bias, limitations of the animal model, and imprecision associated with the results. | 🗹 |
| Generalisability/  translation | | 18 | | Comment on whether, and how, the findings of this study are likely to generalise to other species or experimental conditions, including any relevance to human biology (where appropriate). | 🗹 |
| Protocol registration | | 19 | | Provide a statement indicating whether a protocol (including the research question, key design features, and analysis plan) was prepared before the study, and if and where this protocol was registered. | 🗹 |
| Data access | | 20 | | Provide a statement describing if and where study data are available. | 🗹 |
|  | | 21 | | a. Declare any potential conflicts of interest, including financial and non-financial. If none exist, this should be stated. | 🗹 |
|  | |  | | b. List all funding sources (including grant identifier) and the role of the funder(s) in the design, analysis and reporting of the study. | 🗹 |

The following provides supplementary notes for the table above:

4. Description of Random Allocation of Experimental Animals:

(1) Timing of Randomization：Random allocation was performed after successful induction of the osteoarthritis model and prior to the initiation of drug intervention. This ensured that animals were assigned to groups based on the principle of randomization before applying the primary treatment factor (i.e., administration of different drugs or controls);

(2) Method of Random Sequence Generation：The built-in Random Number Generator function in SPSS Statistics software was used.

To minimize confounding bias, we implemented a three-pronged strategy:

(1) Treatment randomization: Animals were allocated to groups via computer-generated sequences (SPSS v28.0, seed=20230415) post-modeling;

(2) Blinded procedures: Compound preparation/dosing was performed by naive researchers using coded vials, while outcome assessors (behavioral/histological) remained blinded to group identities;

(3) Systematic measurement: All assays were conducted in randomized sample processing order within 48-hour batches.

5. Blinding was maintained throughout key experimental phases:

(1) Allocation: The statistician（Yueying Huang） generating randomization codes (SPSS v21.0) was isolated from animal handling（Xue Jiang）;

(2) Intervention: Pharmacotherapy administrators（Sailiang Zeng） prepared/dosed compounds using coded vials without group knowledge;

(3) Outcome assessment: Behavioral scorers（Zijun Wang）, histopathologists（Yangyang Zhan）, and lab technicians（Lei Lv） processed samples with masked identifiers;

(4) Data analysis: Statisticians（Chun Chen） received de-identified datasets with groups labeled as A-I. Unblinding occurred only after final statistical validation, except for predefined veterinary emergencies (e.g., adverse event investigation).

8. Details of experimental animal usage will be described more accurately in the manuscript: Sprague-Dawley (SD) rats aged 6–7 weeks (200 ± 20 g) of specific pathogen-free (SPF) grade were supplied by Shanghai Regen Biological Co., Ltd. (Experimental Animal Production License: SCXK 2019-0002).
