# Supplementary material for: The Eucommia ulmoides - Achyranthes bidentata pair and their active monomers exert synergistic therapeutic potential for osteoarthritis through the PI3K-AKT pathway
Source: Front Pharmacol. 2025 Aug 11;16:1571884. doi: 10.3389/fphar.2025.1571884 (PMC12375627; doi:10.3389/fphar.2025.1571884)
Supplement: Supplementary file 2 [file Table2.docx]

Tab1 Results of Normality and Homogeneity of Variance Tests for Various Indicators in Crude Water Extract In Vitro Experiments（P-Value）

|  |  | Blank | Model | EU 800 | AB 800 | 200:200 | 400:400 | 800:800 |
| --- | --- | --- | --- | --- | --- | --- | --- | --- |
| NO | Shapiro-Wilk test | 0.1545 | 0.1669 | 0.2634 | 0.2248 | 0.9511 | 0.3862 | 0.4221 |
|  | Brown-Forsythe test | 0.0599 |  |  |  |  |  |  |
| IL-1β | Welch's ANOVA test | 0.0016 | | | | | | |
| IL-6 | Welch's ANOVA test | <0.0001 | | | | | | |
| TNF-a | Welch's ANOVA test | <0.0001 | | | | | | |
| iNOS | Shapiro-Wilk test | 0.4871 | 0.3240 | 0.5824 | 0.7907 | 0.6614 | 0.6188 | 0.4575 |
|  | Brown-Forsythe test | 0.9948 |  |  |  |  |  |  |
| COX-2 | Shapiro-Wilk test | 0.2367 | 0.7745 | 0.9972 | 0.8831 | 0.9595 | 0.7903 | 0.4317 |
|  | Brown-Forsythe test | 0.8046 |  |  |  |  |  |  |
| mmp-3 | Shapiro-Wilk test | 0.3893 | 0.7200 | 0.1716 | 0.5608 | 0.8831 | 0.3993 | 0.6433 |
|  | Brown-Forsythe test | 0.6452 |  |  |  |  |  |  |
| mmp-13 | Shapiro-Wilk test | 0.3503 | 0.7656 | 0.9840 | 0.6412 | 0.7832 | 0.8981 | 0.9092 |
|  | Brown-Forsythe test | 0.9947 |  |  |  |  |  |  |

Tab2 Results of Normality and Homogeneity of Variance Tests for Various Indicators in Crude Water Extract In Vivo Experiments（P-Value）

|  |  | Blank | Model | CL | TH | EU | AB | P-L | P-M | P-H |
| --- | --- | --- | --- | --- | --- | --- | --- | --- | --- | --- |
| IL-1β | Shapiro-Wilk test | 0.1068 | 0.7545 | 0.5423 | 0.8259 | 0.7308 | 0.3572 | 0.2649 | 0.0575 | 0.3130 |
|  | Brown-Forsythe test | 0.7681 |  |  |  |  |  |  |  |  |
| NO | Shapiro-Wilk test | 0.8892 | 0.6287 | 0.4978 | 0.7704 | 0.0874 | 0.2680 | 0.3201 | 0.1800 | 0.8836 |
|  | Brown-Forsythe test | 0.4922 |  |  |  |  |  |  |  |  |
| mmp-3 | Shapiro-Wilk test | 0.4963 | 0.9890 | 0.7061 | 0.1295 | 0.4181 | 0.0637 | 0.4785 | 0.9354 | 0.1626 |
|  | Brown-Forsythe test | 0.5116 |  |  |  |  |  |  |  |  |
| mmp-13 | Welch's ANOVA test | <0.0001 | | | | | | | | |
| Weight | Shapiro-Wilk test | 0.9506 | 0.9739 | 0.9800 | 0.8409 | 0.6759 | 0.2656 | 0.4226 | 0.5719 | 0.4915 |
|  | Brown-Forsythe test | 0.9801 |  |  |  |  |  |  |  |  |
| Paw Withdra  Threshold | Shapiro-Wilk test | 0.9530 | 0.3857 | 0.3870 | 0.7936 | 0.5340 | 0.2386 | 0.1157 | 0.0589 | 0.0990 |
|  | Brown-Forsythe test | 0.0909 |  |  |  |  |  |  |  |  |
| joint diameter | Shapiro-Wilk test | 0.0675 | 0.1353 | 0.4527 | 0.4136 | 0.1742 | 0.8831 | 0.3189 | 0.9893 | 0.4341 |
|  | Brown-Forsythe test | 0.7313 |  |  |  |  |  |  |  |  |
| mmp-3（protein） | Shapiro-Wilk test | 0.1646 | 0.8126 | 0.5241 | 0.9900 | 0.7171 | 0.8017 | 0.8921 | 0.8227 | 0.2405 |
|  | Brown-Forsythe test | 0.2749 |  |  |  |  |  |  |  |  |
| mmp-13  （protein） | Shapiro-Wilk test | 0.4861 | 0.4821 | 0.2606 | 0.7794 | 0.8764 | 0.9019 | 0.1422 | 0.9580 | 0.7533 |
|  | Brown-Forsythe test | 0.8324 |  |  |  |  |  |  |  |  |

Tab3 Results of Normality and Homogeneity of Variance Tests for Various Indicators in Monomeric Compound In Vitro Experiments（P-Value）

|  |  | Blank | Model | CHI | PIN | CP |
| --- | --- | --- | --- | --- | --- | --- |
| iNOS-chro | Shapiro-Wilk test | 0.7804 | 0.2196 | 0.9644 | 0.4974 | 0.9352 |
|  | Brown-Forsythe test | 0.7313 |  |  |  |  |
| mmp-13 | Welch's ANOVA test | <0.0001 | | | | |
| mmp-3 | Welch's ANOVA test | <0.0001 | | | | |
| IL-1β | Shapiro-Wilk test | 0.1572 | 0.6878 | 0.4156 | 0.5368 | 0.9153 |
|  | Brown-Forsythe test | 0.4506 |  |  |  |  |
| iNOS -adtc | Shapiro-Wilk test | 0.5275 | 0.1736 | 0.6878 | 0.1650 | 0.7740 |
|  | Brown-Forsythe test | 0.7488 |  |  |  |  |
| mmp-3 | Welch's ANOVA test | 0.0001 | | | | |
| mmp-13 | Shapiro-Wilk test | 0.4974 | 0.3721 | 0.8168 | 0.8105 | 0.9296 |
|  | Brown-Forsythe test | 0.9602 |  |  |  |  |
| IL-1β | Shapiro-Wilk test | 0.7391 | 0.8061 | 0.3172 | 0.9702 | 0.4173 |
|  | Brown-Forsythe test | 0.8268 |  |  |  |  |
| AKT | Welch's ANOVA test | 0.0395 | | | | |
| p-AKT | Shapiro-Wilk test | 0.4280 | 0.3409 | 0.0173 | 0.8178 | 0.7751 |
|  | Brown-Forsythe test | 0.7798 | | | | |
| p-Akt/AKT | Shapiro-Wilk test | 0.1744 | 0.1418 | 0.9425 | 0.6454 | 0.8870 |
|  | Brown-Forsythe test | 0.4864 | | | | |
| BAX | Shapiro-Wilk test | 0.9051 | 0.2351 | 0.2822 | 0.4642 | 0.9013 |
|  | Brown-Forsythe test | 0.8582 | | | | |
| Caspase3 | Welch's ANOVA test | 0.0150 | | | | |
| Caspase9 | Shapiro-Wilk test | 0.0901 | 0.3995 | 0.8402 | 0.3359 | 0.6565 |
|  | Brown-Forsythe test | 0.4097 | | | | |
